# Short-Term Stability in Refractive Status Despite Large Fluctuations in Glucose Levels in Diabetes Mellitus Type 1 and 2

**DOI:** 10.1371/journal.pone.0052947

**Published:** 2012-12-28

**Authors:** Byki Huntjens, W. Neil Charman, Helena Workman, Sarah L. Hosking, Clare O’Donnell

**Affiliations:** 1 Division of Optometry and Visual Science, City University London, London, United Kingdom; 2 Faculty of Life Sciences, The University of Manchester, London, United Kingdom; 3 School of Life and Health Sciences, Aston University, Birmingham, United Kingdom; 4 Department of Ophthalmology, University of Melbourne, Melbourne, Australia; 5 Optegra Eye Sciences, Manchester, United Kingdom; Univeristy of Melbourne, Australia

## Abstract

**Purpose:**

This work investigates how short-term changes in blood glucose concentration affect the refractive components of the diabetic eye in patients with long-term Type 1 and Type 2 diabetes.

**Methods:**

Blood glucose concentration, refractive error components (mean spherical equivalent MSE, J0, J45), central corneal thickness (CCT), anterior chamber depth (ACD), crystalline lens thickness (LT), axial length (AL) and ocular aberrations were monitored at two-hourly intervals over a 12-hour period in: 20 T1DM patients (mean age ± SD) 38±14 years, baseline HbA1c 8.6±1.9%; 21 T2DM patients (mean age ± SD) 56±11 years, HbA1c 7.5±1.8%; and in 20 control subjects (mean age ± SD) 49±23 years, HbA1c 5.5±0.5%. The refractive and biometric results were compared with the corresponding changes in blood glucose concentration.

**Results:**

Blood glucose concentration at different times was found to vary significantly within (p<0.0005) and between groups (p<0.0005). However, the refractive error components and ocular aberrations were not found to alter significantly over the day in either the diabetic patients or the control subjects (p>0.05). Minor changes of marginal statistical or optical significance were observed in some biometric parameters. Similarly there were some marginally significant differences between the baseline biometric parameters of well-controlled and poorly-controlled diabetic subjects.

**Conclusion:**

This work suggests that normal, short-term fluctuations (of up to about 6 mM/l on a timescale of a few hours) in the blood glucose levels of diabetics are not usually associated with acute changes in refractive error or ocular wavefront aberrations. It is therefore possible that factors other than refractive error fluctuations are sometimes responsible for the transient visual problems often reported by diabetic patients.

## Introduction

The effects of diabetes mellitus (DM) on the anterior structures of the eye are less frequently reported than its effects on the retina. However, anterior ocular changes can also lead to visual problems. The bulk of the relevant literature describes changes in refractive error due to fluctuating blood glucose levels. These typically occur soon after the onset of treatment for diabetes [1], [2], [3], [4], [5]; they may be either acute (short-term) or chronic (long-term), and either myopic [1], [2], [3], [6] or hyperopic [4], [5], [7].

It has been suggested that diabetes-induced changes occurring in the aqueous humour [8], [9], cornea [10], [11], [12] and crystalline lens [13], [14], [15] could play a key role in refractive fluctuations. It is hypothesized that glucose enters the crystalline lens via the aqueous humour by a process of facilitated diffusion. Some experimental studies suggest that, in diabetes, hyperglycemia leads to an excessive uptake of glucose into the lens cells and fibres, which activates alternative routes for glucose handling such as the aldose reductase pathway. This instigates intracellular accumulation of sorbitol [16] followed by lenticular swelling [17], which causes a myopic shift. During hyperglycemia, the increased flux of glucose through the polyol pathway accounts for as much as one-third of the total glucose turnover [18], [19]. Aldose reductase-induced osmotic stress seems to be the cause of diabetic cataract [20]. Conversely, a decrease in glucose concentration in the aqueous humour is predicted to change the osmotic pressure between the aqueous humour, lens and vitreous humour with a decrease in the refractive index of the lens, leading to a hyperopic refractive shift [21]. The exact effects are likely to be complex and to vary with the individual patient, since the power of the lens will be affected by any changes in thickness, surface curvature and gradient of refractive index, and these changes will depend on the individual’s age and their response to such factors as the activity of aldose reductase in the lens epithelial cells [22].

Most studies have examined the effects on refractive error of changing blood glucose levels after insulin treatment has been instigated for the first time in newly-diagnosed diabetic patients [2], [4], [5], [7]. Several studies involve small selected subsets of patients who have recently complained of visual blur, rather than typical, unselected, diabetic individuals [23], [24]. However, such work may not reflect the response of the eye to the more typical glucose fluctuations that are experienced by long-term diabetic patients on a day-to-day basis. To the best of our knowledge, such data are not readily available. Rubin *et al.* [25] investigated the diurnal variation in refractive errors in one diabetic patient versus one non-diabetic subject using an autorefractor, but found no clinically significant differences between the subjects. Agardh *et al.* [26] found a stable refraction and visual acuity in fifty-three diabetic patients at different occasions within a month. Since blood glucose levels in diabetic patients are known to fluctuate significantly during the day, the aim of the present study was to compare in detail the short-term daily variation in the refraction, ocular aberration and ocular components in diabetic patients with those of control subjects. We found no significant direct correlation between fluctuating blood glucose levels and refractive error or ocular biometry in the diabetic eyes.

## Methods

This prospective, controlled study was conducted at a single clinical site (Optegra Birmingham Eye Hospital, formerly known as The Aston Academy, Life Sciences, Aston University, Birmingham, United Kingdom). Diabetic patients and control subjects were recruited from an advertisement placed in a local newspaper and from a University public access clinic and student and staff populations.

### Ethics Statement

Ethical approval was obtained from the Institutional Research Ethics Committee and informed consent was obtained from all subjects, conforming to the tenets of the Declaration of Helsinki.

All subjects attended the clinic between 0800 and 2000 hours, data being collected at approximately two-hourly intervals (0800, 1030, 1230, 1500, 1700, and 1900 hours). Subjects were screened to exclude those who had previous ocular surgery, amblyopia, Snellen acuity worse than 6/9, or past invasive treatment for diabetic maculopathy, this being checked using non-mydriatic fundus photography (Canon CR-DGi fundus camera; Canon USA, Inc. New York, USA) and optical coherence tomography (Stratus OCT; Carl Zeiss Meditec AG, Jena, Germany). Cataract was defined as either ‘present’ or ‘not present’ following slit lamp examination. Cataract was graded using the Lens Opacities Classification III System [27]. We classified subjects as having no clinically significant cataract (NO1 or NC1, C1, PS1) or clinically significant cataract (all others) for the purpose of analysis. The classification of diabetic retinopathy [28] was determined using non-mydriatic fundus photography and direct ophthalmoscopy was performed to confirm the results.

Previously-experienced blurred vision during hypo- or hyperglycemia was recorded but not considered as an inclusion criterion. Contact lens wearers were asked to use their spectacles on the day of the study and to suspend contact lens wear on the previous day, to minimise the influence of any short-term corneal changes induced by contact lens wear. The right or the left eye of each subject was randomly selected for the clinical assessments. Subjects were encouraged to eat, drink and use their medications as they normally would. Breakfast was taken between the first and the second measurement session, lunch between the third and fourth session, and dinner between the fifth and sixth set of measurements.

Measurements of objective refraction and ocular aberrations for a 6 mm pupil were carried out using an OPD ARK-10000 autorefractometer (Nidek Co., Ltd., Tokyo, Japan). Axial length and anterior chamber depth were measured using an IOLMaster (Carl Zeiss Meditec AG, Jena, Germany). Lens thickness measurements were performed using an Echorule Ultrasonic Biometer (Vision Care/Phakosystems Inc., Downsview, Canada) with one drop of topical anaesthetic (oxybuprocaine hydrochloride 0.4% w/v; Chauvin Pharmaceuticals Ltd, Kingston-upon-Thames, UK), and corneal thickness measurements were obtained using a Pachmate DGH-550 Pachette 2 (DGH Technology Inc, Exton, USA) pachymeter, using the same type of anaesthetic. All the above measurements were repeated 3 times at each time point (except for pachymetry, where 5 measurements were taken) and the mean was used for data analysis.

Intersession coefficients of repeatability (COR), given as ±1.96× standard deviation of the differences between two sets of measures taken on the same subjects, have been reported for the techniques used in the present study. They are: 18.0 µm for central corneal thickness measured using ultrasound pachymetry [29]; 0.049 mm and 0.043 mm respectively for anterior chamber depth and axial length measured using the IOLMaster [30]; and 0.196 mm for lens thickness obtained using ultrasound biometry [31]. The Nidek OPD autorefractometer has a COR of 0.7–0.8 D for the vertical and horizontal meridians [32]. Fluctuations in any of our ocular parameters are only considered to be clinically significant if they are found to be larger than the COR values.

Information relating to long-term diabetic metabolic control was obtained from an HbA1c test (blood taken from the forearm by a nurse and analysed using a laboratory-based Roche Serum Work Area glucose analyser), and short-term blood glucose concentration taken from a finger was assessed at each measurement session using a HemoCue 201+ capillary blood test (HemoCue AB Ängelholm, Sweden).

Fundus photography and the HbA1c blood test were performed in the diabetic patients at baseline only. Autorefraction, aberrometry, axial length and anterior chamber depth, corneal thickness, lens thickness and blood glucose measurement were repeated approximately every two hours in all diabetic patients and control subjects.

### Statistical Analysis

Manifest auto-refraction in conventional script notation (sphere S, cylinder C, and axis θ, nominally measured to 0.01D and 1 degree) was converted to power vector coordinates as described by Thibos *et al*. [33]:

1. Mean spherical equivalent (MSE), using

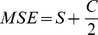

1. Orthogonal component 90 to 180 degrees of astigmatism (J0), given by

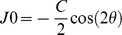

1. Orthogonal component 45 to 135 degrees of astigmatism (J45), using

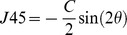



All statistical analyses were performed using SPSS version 19.0 for Windows (SPSS Inc., Chicago, USA). Kolmogorov-Smirnov tests revealed no significant deviation from a normal distribution for most of the test parameters (BGLs, CCT, ACD, LT, AL, MSE, J180, J45 and vertical coma) at baseline (p>0.05). Due to their statistically significant Kolmogorov-Smirnov results, some ocular aberrations (horizontal coma, spherical aberrations, and higher-order RMS) were analysed using non-parametric tests (i.e. Mann-Whitney U test, Kruskal-Wallis test, and Spearman Rank Order Correlation for baseline measurements, and Friedman test to investigate 12-hour fluctuations).

**Table 1:** Patient demographics.

	T2DM patients	T1DM patients	Control subjects	p-value
**Subjects**	21	20	20	
(Number)				
**Age**	56±11	38±14	49±23	0.004*
(Mean ± SD in years)				
**Gender**	12∶9	9∶11	11∶9	0.7
(male: female)				
**Ethnicity**	18∶2:1	17∶1:2	18∶2:0	0.9
(Caucasian: Asian: Black)				
**Eye**	12∶9	10∶10	11∶9	0.9
(right: left)				
**Cataract**	6∶15	3∶17	6∶14	0.5
(present: not present) [27]				
**Diabetic retinopathy**	9∶10:2	10∶5:5	–	0.7
(none: background: pre-proliferative) [28]				
**Duration of the disease**	12±8	23±15	–	0.007*
(Mean ± SD in years)				
**HbA1c level**	7.5±1.8	8.6±1.9	5.5±0.5	0.07 (DM only)
(%)				<0.0005* (DM+control)
**Diabetic control**	14∶7	4∶16	–	0.07
(HbA1c ≤7.5 mM/l (135 mg/dl): HbA1c >7.5 mM/l)				
**Glucose levels**	8.3±3.0	11.1±5.0	4.6±0.5	<0.0005*
mM/l (mg/dl)	(149±54)	(200±90)	(83±9)	
*HemoCue range: 0–22.2* *mM/l*				
**Diabetic medication**	7∶6:7∶1	20∶0:0∶0	–	
(insulin: tablets: both: diet only)				

Where data for three groups are given, P values refer to probabilities that the parameter differs significantly between the 3 groups, otherwise the probabilities refer to differences between the two diabetic groups.

* indicates a statistically significant p-value of ≤0.05.

Each variable was first assessed for between-group differences at baseline. Repeated-measures ANOVA were used to investigate the variation in parameters over the course of the day. Post hoc analysis identified any session that showed significant differences compared to other sessions, or any significant inter-group differences. For analysis of horizontal coma, spherical aberrations, and higher-order RMS data, a non-parametric Friedman test was performed. Additionally, stepwise multiple regression models were used to explore possible relationships between each test parameter and a number of potential predictor variables for each of the six sessions during the day. Two models were designed to explore differences between the two diabetic groups and the diabetic versus the control groups separately. The predictor variables of model 1 were age, duration of the disease, diabetic retinopathy status (using a classification based on the Early Treatment Diabetic Retinopathy Study [34]), and blood glucose levels; model 2 included age, disease status (T1DM or T2DM), HbA1c and blood glucose levels. A p-value of ≤0.05 was taken to indicate statistical significance for baseline measurements, while a p-value of ≤0.01 represented statistical significance in all repeated measures and multiple regression tests.

## Results

### Baseline Data

Data on non-optical parameters are given in Table 1. The T1DM patients were significantly younger than T2DM patients and the control group (p = 0.004). The control group had significantly lower blood glucose levels (p<0.0005). No statistically significant differences were found between the groups for gender, ethnicity, or the presence of cataract [27] (p>0.05).

Between the diabetic groups, the duration of the disease was greater in the T1DM patients (p = 0.007). On the basis that an HbA1c reading of ≤7.5% indicated reasonably ‘good’ diabetic metabolic control [35], whereas HbA1c >7.5% was indicative of ‘poor’ metabolic control, the proportion of T1DM with higher HbA1c readings was greater than in the T2DM group, although the difference failed to reach statistical significance (p = 0.07).

All the baseline data for patients in the two diabetic groups were analysed in terms of whether their metabolic control was “good” or “poor” as defined above. Table 2 shows that the diabetic patients in the well-controlled group had a mean HbA1c of 6.5±0.7%, while the mean HbA1c value in the poorly-controlled group was 9.2±1.7% (p<0.0005). Considering the optical and biometric data (Table 2), patients with poorly-controlled diabetes showed a significantly greater central corneal thickness CCT (t(39) = −2.03; p = 0.049), smaller anterior chamber depth ACD (t(39) = 2.73; p = 0.006), and smaller axial length AL (t(39) = 2.28; p = 0.03) compared to those with well-controlled diabetes. The shallower anterior chambers of the poorly-controlled diabetic patients were accompanied by thicker lenses (even though the patients were, on average, younger), but the difference in LT compared with the well-controlled patients was not statistically significant. Except for J45, which showed a higher mean value in poorly-controlled compared to well-controlled diabetic patients (p = 0.03), no differences between these two groups were found for refractive components, individual aberrations, or total RMS wavefront error.

**Table 2:** Baseline data for well-controlled (HbA1c ≤7.5%) and poorly-controlled (HbA1c >7.5%) diabetic patients (including both T1DM and T2DM) for all ocular parameters.

	Well-controlled	Poorly-controlled	p-value
**Subjects** (number)	18	23	
**HbA1c** (mean ± SD in %)	6.5±0.7	9.2±1.7	<0.0005*
**Age** (mean ± SD in years)	54±14	42±15	0.01*
**MSE** (mean ± SD in Diopters)	−0.63±2.28	0.033±2.44	0.38
**J0** (mean ± SD in Diopters)	−0.02±0.28	−0.02±0.31	0.99
**J45** (mean ± SD in Diopters)	−0.15±0.26	0.04±0.28	0.03*
**CCT** (mean ± SD in microns)	535±36	560±42	0.049*
**ACD** (mean ± SD in mm)	3.28±0.24	3.05±0.32	0.01*
**LT** (mean ± SD in mm)	4.12±0.70	4.45±0.56	0.13
**AL** (mean ± SD in mm)	23.83±1.07	23.03±1.15	0.03*
**Horizontal coma** (mean ± SD in microns)	−0.34±0.19	−0.12±0.18	0.38
**Vertical coma** (mean ± SD in microns)	0.05±0.13	−0.04±0.43	0.58
**Spherical aberrations** (mean ± SD in microns)	0.21±0.41	0.03±0.15	0.21
**RMS error** (mean ± SD in microns)	0.58±0.25	0.69±0.99	0.78

Aberrations were measured for 6 mm pupils.

* indicates a statistically significant p-value of ≤0.05.

When data for all the diabetic and control subjects were pooled, age was found to have a significant effect on ACD and LT. ACD was found to become shallower with age (F(2,58) = 5.22; p = 0.008), and LT was found to increase with age (F(2,58) = 5.03; p = 0.01). The MSE values in diabetic and control subjects ranged from –9.63D to 8.75D. Post-hoc analysis among the different age groups revealed higher levels of myopia in younger subjects (<30 years) compared to older subjects (>60 years) (F(2,58) = 5.42; p = 0.006). Subjects in the age-range 31–59 years did not show a significant difference in MSE compared to the <30 or >60 year old subject groups (p>0.05). There were no differences in the amount of oblique astigmatism for sub-groups split by gender, age or by the degree of diabetic retinopathy present. However, posthoc analysis revealed an increased J45 value in T1DM compared to T2DM patients (p = 0.005). A significant positive correlation was found between the J45 value and the duration of disease (r = 0.425; p = 0.006).

### 12-hour Fluctuations

**Figure 1:**
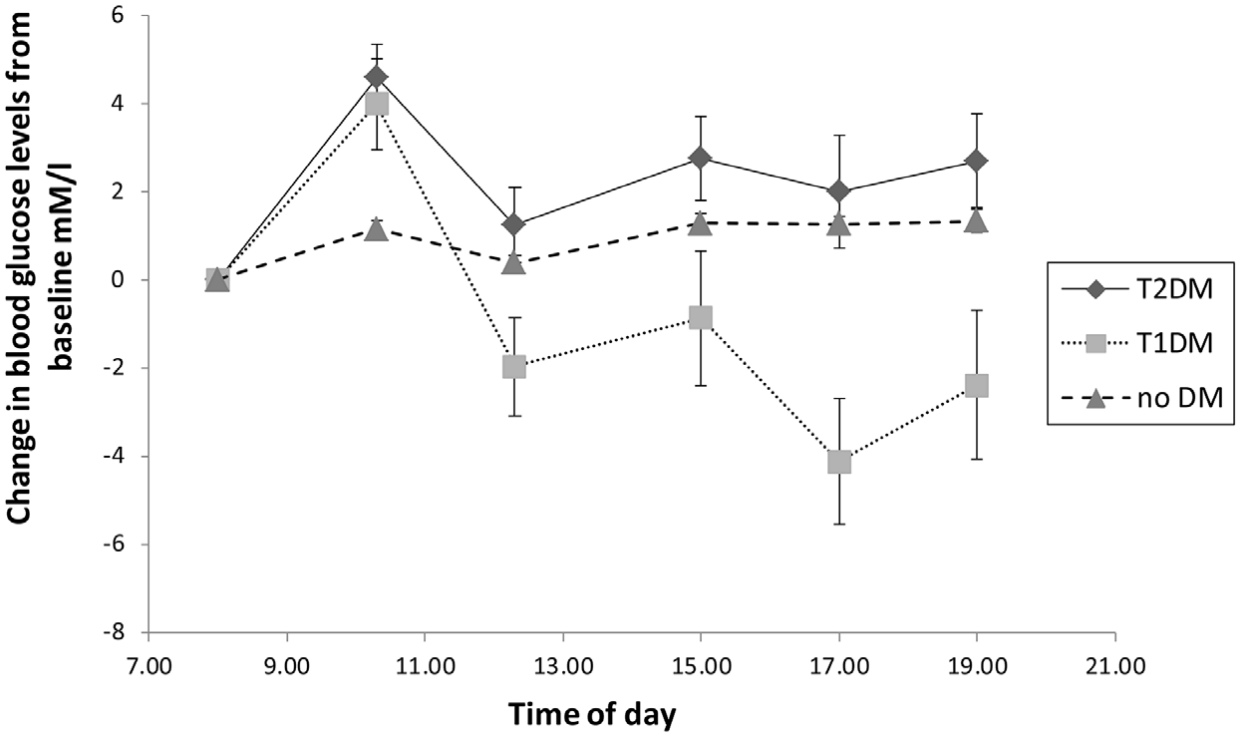
Change in blood glucose levels from baseline over time for each group. T2DM are represented as diamonds; T1DM are represented as squares; control subjects are represented as triangles. The error bars represent 1 standard error of the mean (SD/√n). Subjects ate their meals around 0900, 1330 and 1730 hours. Mean baseline blood glucose levels as determined with the Hemocue test were 11.1±5.0 mM/l (200±90 mg/dl) for the T1DM; 8.3±3.0 mM/l (149±54 mg/dl) for T2DM; and 4.6±0.5 mM/l (83±9 mg/dl) for the control subjects.

As expected, blood glucose levels were found to vary significantly within groups (p<0.0005) and between groups (p<0.0005). The short-term changes in blood glucose concentration for the three groups are shown in Figure 1. Since the variance in blood glucose levels at each session was large in the diabetic groups, the error bars show ±1 standard error of the mean (SEM = SD/√n). The largest changes, of up to about 6 mM/l, occur in T1DM, the smallest in the controls. As the mean baseline blood glucose levels are also highest for the T1DM group and smallest for the controls (Table 2), the differences between the groups are reduced if the changes are normalised in terms of the baseline values (i.e. normalised change = absolute change/baseline level).

**Figure 2:**
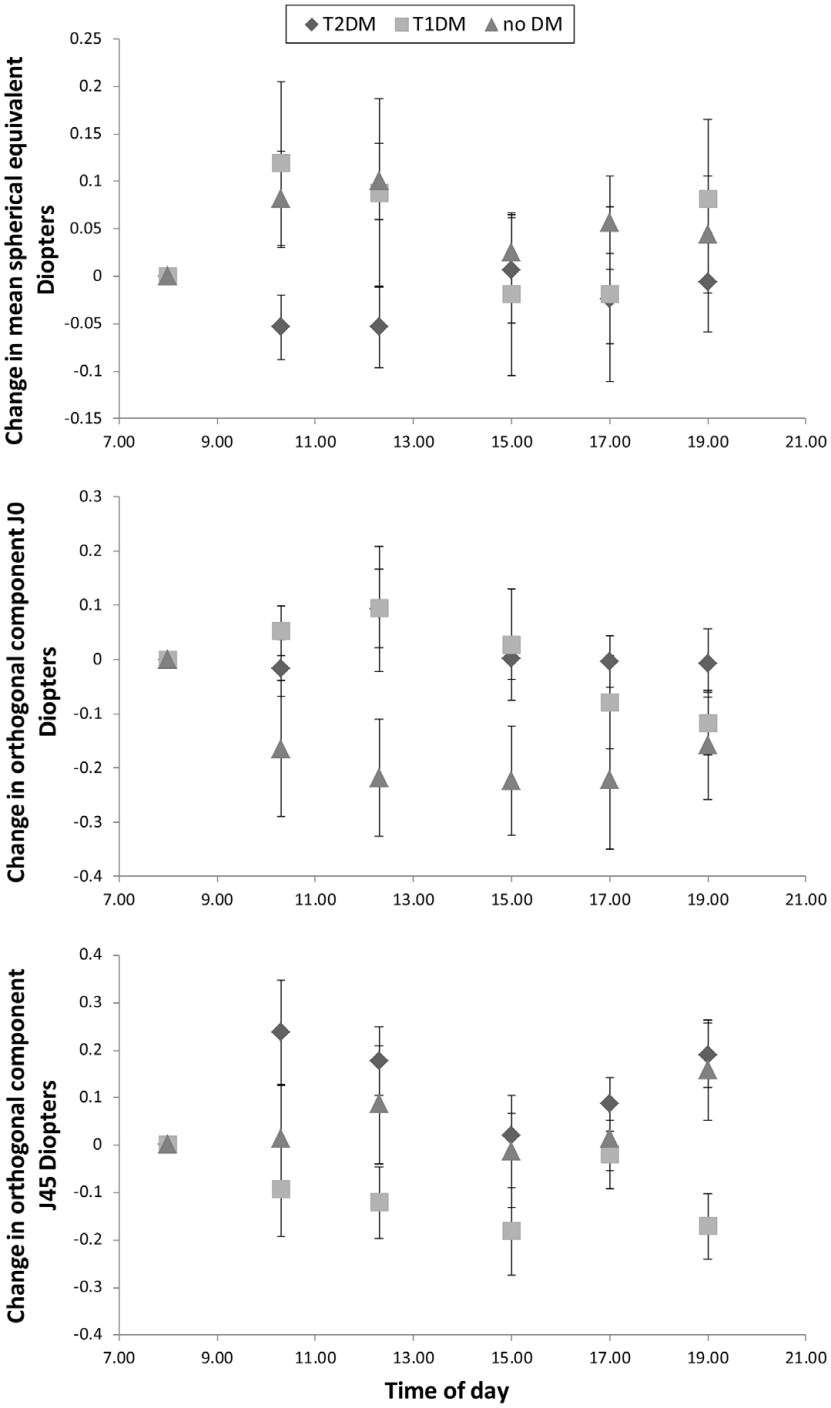
Mean changes over time in the components of refractive error (MSE, J0, and J45) from baseline measurements for each group. T2DM are represented as diamonds; T1DM are represented as squares; control subjects are represented as triangles. The error bars show ± SEM.

**Figure 3:**
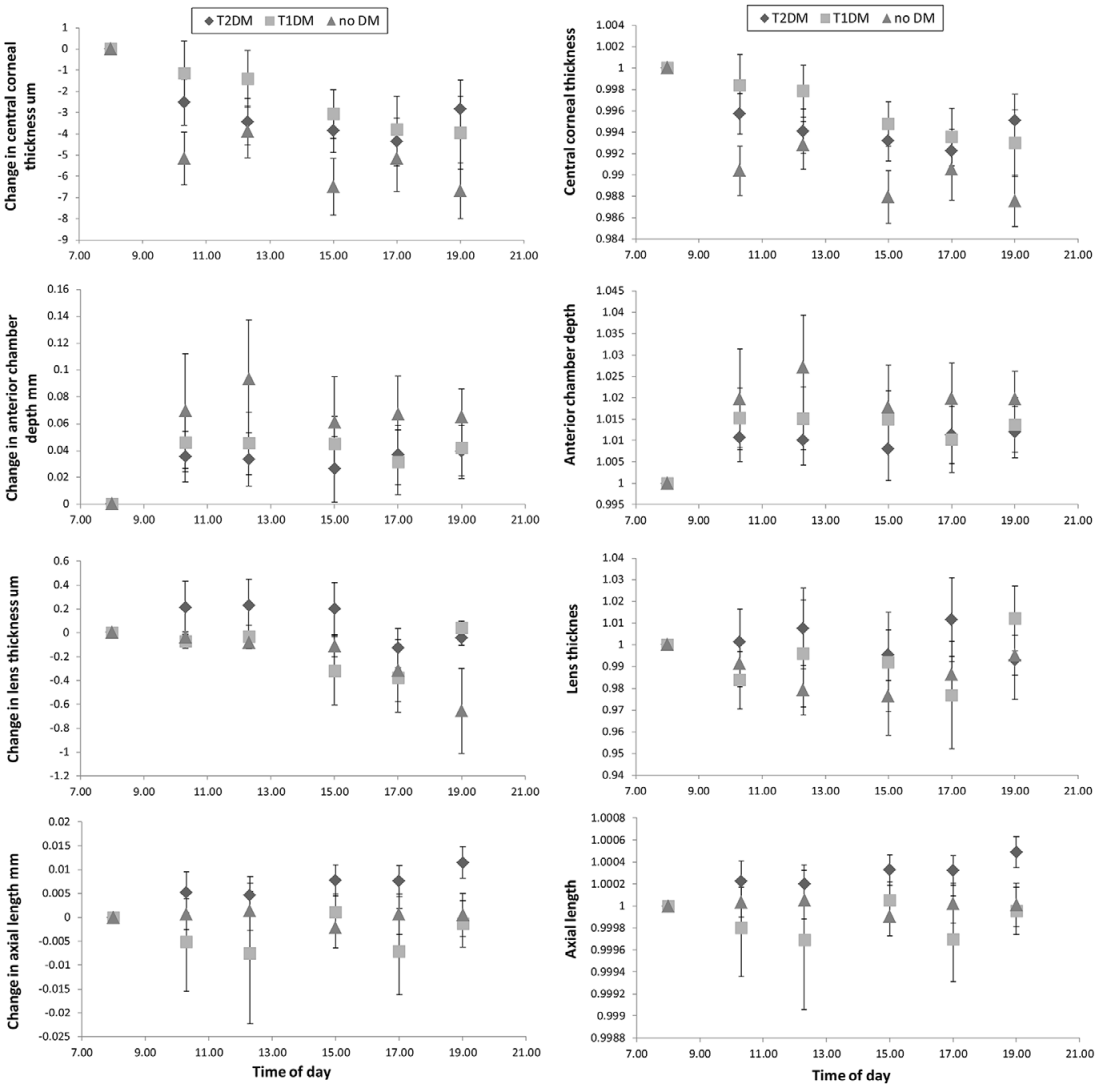
Mean change from baseline measurements for ocular parameters CCT, ACD, LT, and AL over time for each group (A) and the relative value of each parameter (i.e. the absolute value of the parameter divided by its baseline value) as a function of time (B). T2DM are represented as diamonds; T1DM are represented as squares; control subjects are represented as triangles. The error bars show ± SEM.

Data for the 12-hour variation in the components of refractive error (MSE, J0, and J45) are shown in Figure 2, plotted in terms of the change from the baseline value of the parameter. All dioptric changes are small and close to the limits set by the reliability of the measurement equipment used (but note that since J0 and J45 are cross-cylinders, the changes in cylindrical correction C are twice those in J0 and J45). Changes in several of the key biometric parameters are presented in Figure 3A as departures from their baseline values. Figure 3B gives the data in a different form, the absolute values of each parameter at each point in time being normalised in terms of the baseline value of the parameter. Again, the magnitudes of any of the changes observed are close to the limits set by the coefficients of repeatability of the instruments used (see Methods section). With this proviso, several variables showed small but statistically significant differences either with time (for all subjects) or between the three groups (T1DM, T2DM, or control). Post-hoc analysis showed that the CCT was higher at baseline compared to at all other times during the day (t-test; p<0.0005), the ACD was significantly shallower at baseline (0800 hours) compared to at 1230, 1700, and 1900 hours (t-test; p<0.003). T2DM patients showed a longer AL in the evening (1900 hours) compared to that measured at baseline (p = 0.002) and T1DM had a shorter AL in the evening (1900 hours) compared to that measured around midday (1230 hours) and the afternoon (1700 hours) (p<0.0005 and p = 0.001 respectively). No significant change in AL was found throughout the day in the non-diabetic control subjects. Considering inter-group differences, LT showed a significant difference in fluctuation over the 12-hour period between groups (p = 0.007). A consistently thicker lens compared to baseline was found in T2DM versus control subjects (Tukey’s HSD; p = 0.006). There was no statistically significant difference between the LT variation in T1DM compared to T2DM (p = 0.42), or T1DM versus control subjects (p = 0.11).

### Multiple Regression Analysis

Multiple regression models were used to investigate the possible influence of the predictor variables age, disease status, duration of the disease, blood glucose levels, HbA1c levels, and diabetic retinopathy on the biometric study parameters. Both models were applied to each measurement session separately and analysis was performed to ensure there was no violation of the assumptions of normality and linearity. If the predictor variable showed a statistically significant result at 3 or more out of the 6 sessions, it was considered that the predictor variable had a significant influence on the biometric parameter of interest. These analyses revealed that an increase of 4.5 mM/l (81 mg/dl) in blood glucose levels corresponded with an increase of 10 µm in CCT in the diabetic patients and control subjects. On average, ACD reduced by approximately 0.07 mm for every 10 years of age. However, when diabetic patients were studied separately, ACD decreased by 0.13 mm for every 10 years the patient had diabetes. Additionally, an increase of 2% in HbA1c corresponded to a decrease in the ACD of 0.12 mm. The model predicted that LT increases by approximately 0.13 mm for every 10 years of age for all subjects. In diabetic patients, the LT increased by around 0.24 mm for every 10 years of duration of disease, and an increase of 2% in HbA1c levels corresponded to an increase in LT of 0.21 mm. The mean spherical equivalent showed an increase of 0.52D every 10 years of age for all subjects, while no difference was found between the diabetic and non-diabetic subjects. No relationships with any of the predictor variables were found for AL, J0, J45, and ocular aberrations.

## Discussion

Changes in refractive error in uncontrolled or undiagnosed diabetic patients have often been reported, although there is uncertainty about the precise mechanism of such changes. Much of the literature involves patients who have only just commenced treatment [2], [4], [5], [7], [8], [36] or who have recently reported that their vision had become blurred [23], [37].

The present study investigated changes in the refractive optics of the eyes in long-term diabetic patients and control subjects over a period of 12 hours, in relation to normal acute, non-fasting, short-term changes in blood glucose levels. It was hypothesised that with increasing blood glucose levels the refractive error of diabetic eyes would alter, possibly towards myopia, while the non-diabetic subjects’ refractive error was expected to stay more or less constant over the course of the day. Additionally, we hypothesised that diabetic patients would show greater short-term variation in the anterior ocular parameters than control subjects, and possibly larger changes in refractive indices of these ocular components, these leading to any refractive changes observed. Although we found marginally significant baseline differences in anterior chamber depth in poorly-controlled compared to well-controlled diabetic patients, the mean changes in the refractive parameters throughout the day in the diabetic patients were clinically insignificant (<0.25 D in MSE) and similar to those in the non-diabetic subjects. Similarly, there was no evidence that ocular aberrations were systematically higher at baseline in diabetic patients as compared to controls, or that any aberration (horizontal or vertical coma, spherical aberrations, or RMS error) showed greater temporal variation in the diabetic groups.

The absence of significant short-term refractive changes appears, at first sight, to be incompatible with the results of one of the few studies to have investigated acute ocular changes with fluctuating blood glucose levels. Although Wiemer *et al.* found no significant changes in the shape of the cornea or lens in 25 diabetic patients after hyperglycemia [37]; nine of their patients showed small but significant hyperopic or myopic shifts. However, in contrast to our study, Weimer *et al.*’s patients returned for the second visit on another day (average 51 days later) and their results could therefore refer to longer-term rather than true acute ocular changes. Moreover, their patients were selected on the basis that they initially complained of blurred vision. A different study conducted by the same authors [24] revealed no changes in refractive properties after acute (30 minute intervals) changes in blood glucose levels. However, the subjects in this study had been rendered ‘diabetic’ by somatostatin injection which raised the blood glucose level by around 15 mM/L. This approach has been used previously for similar research purposes [1], [38] but it is not clear whether it adequately simulates the behaviour of real diabetic patients [1], [39]. Similarly, as noted earlier, most of the studies reporting marked refractive change involve patients who have either just commenced treatment or who have recently reported that their vision had become blurred. Thus the composition of our patient groups is substantially different (and possibly more representative of the general population of diabetics) which may partly explain the different results found.

It is also helpful to consider here the timescales of possible changes in blood sugar level and refraction as revealed by previous studies. Okomoto *et al.* [4] found that patients whose marked initial hyperglycaemia (blood glucose level ≥22 mM/l) was being brought under control (to around 6 mM/l) typically experienced a hyperopic refractive shift which peaked between 3 and 30 days after commencement of treatment. The delay and the magnitude of the refractive shift increased with the initial blood glucose level. Refractive recovery times extended from about 12 to 90 days, with the longest times being associated with the largest initial refractive changes. Patients with an initial blood glucose level of 22 mM/l typically showed hyperopic shifts of only about 0.6 D, the peak of this change being reached about 7 days after commencement of treatment and decaying to zero after about 28 days. Broadly similar results were found by Giusti [2]. The modest, and relatively slow, refractive changes in response to changes in blood glucose levels of about 16 mM/l suggest that it is not surprising that our much smaller and more transient changes in blood glucose (a maximum of around 6 mM/l for the T1DM group on a timescale of a few hours, see Figure 1) failed to cause any detectable refractive change. It would appear to be reasonable to suppose that the time constants of any processes affecting the gradients of refractive index within the lens are too long for small, short-term changes in blood glucose to have any significant effect on refraction.

A clear limitation of the present study is the relatively small number (41) of diabetic patients involved. While our finding of minimal refractive or aberrational change in the presence of normal, modest, diurnal fluctuations in blood sugar level may be typical of such patients, we cannot rule out the possibility that, in a larger population, some individuals may display marked acute changes in either or both blood sugar and refraction. None of our patients complained of blurred vision during the measurement period and it may be that, had our patients been selected on the basis of such complaints, refractive changes might have been observed [37]. A further limitation is the substantial range in age and duration of disease in our diabetic groups, which might be expected to increase the variation across subjects and make subtle changes more difficult to detect: we found it difficult to recruit more homogeneous groups willing to undertake the lengthy set of measurements involved in the study.

While no significant diurnal changes were found in the vector components of refraction, the variations in other optical and biometric parameters are of interest. Short-term variations in CCT have been described previously in non-diabetic eyes [40], [41]. Like the present study and despite differences in instrumentation used and time course studied, these previous studies show that the cornea is thickest immediately after awakening [40], [42]. In our study the short-term variation in CCT (around 5 µm) was similar in the control subjects and the diabetic patients and, even though the diabetic corneas were thicker compared to the controls, there were no significant differences between the three groups at baseline. This suggests that short-term changes in blood glucose levels do not have a significant effect on the CCT in diabetic patients. However, any difference in short-term CCT variation between the diabetic and non-diabetic corneas could have been masked by the decreased corneal deswelling response known to occur in diabetic patients [43], [44]. This could also explain the significantly thicker corneas in poorly-controlled compared to well-controlled diabetic patients. Additionally, our results showed that the ACD was significantly decreased in poorly-controlled diabetic subjects compared to the well-controlled group. This does not appear to have been reported previously. It is of interest that ACD increased after baseline in all subjects, by around 50 µm. It may be that this is due to a posterior movement of the anterior surface of the lens following a slight relaxation of accommodation: the measurements of LT are not sufficiently sensitive to confirm this. No previous study has reported short-term variation in LT and our data also failed to show significant differences in fluctuation in the LT during the day in the diabetes versus the control subjects (p = 0.86). Since it is difficult to completely control accommodation using A-scan biometry, it was concluded that small changes in lens thickness or curvatures, which might occur in diabetic patients, could only be measured clinically if (micro-) fluctuations in accommodation were eliminated.

Over the 12-hour period, a significant increase in AL occurred in T2DM patients with the greatest value being measured at 1900 hours. The control subjects did not show a significant short-term variation in AL, although these subjects did show an increased AL in the middle of the day, in agreement with Stone *et al.* [45] Our results also show that the mean absolute maximum change in AL during the day was 13 µm in T2DM (p = 0.002); 14 µm in T1DM (p = 0.01); and 14 µm in the control subjects (p = 0.01). All these figures are at the limits of the resolution of the IOLMaster. However, if the optical power of the eye remains constant, a 1 mm shift in the AL from the cornea to Bruch’s membrane corresponds to about 2.7D of optical defocus [46]. As a result, the mean short-term AL fluctuation, 14 µm, would correspond to a short-term shift of only ±0.038D. Assuming that the depth-of-focus of the human eye is approximately 0.3D [46], this short-term variation in AL is too small to be appreciated subjectively or measured clinically. The observed increase in AL at the last session in T2DM compared to baseline could not have been induced by corneal thickness changes, as the profiles do not overlap. Possible reasons for the difference, if any, in the short-term cycle of AL measurements between T1DM and T2DM patients could be nutritional (carbohydrate intake), as previously suggested by Stone *et al.* [45].

As already remarked, none of our diabetic patients complained of marked changes in the clarity of their vision during the limited 12-hour test period. Nevertheless the possibility that vision may become degraded in the absence of optical change [37], perhaps due to neural factors, is an interesting one. Further studies in which temporal changes in visual performance, in terms of, e.g., visual acuity or contrast sensitivity, are measured at the same time as blood sugar levels and the optical characteristics of the eye should help to clarify this question.

### Conclusions

Contrary to our expectations, this study showed that, in the relatively small (41) number of long-term diabetics studied, typical short-term (12 hours) fluctuations in blood glucose levels did not induce clinically-detectable short-term changes in refractive error, ocular aberrations or the anterior ocular biometric parameters. Nevertheless, some diabetic patients do complain about their vision during hypoglycaemia and hyperglycaemia and previous studies of the subset of patients with such complaints have demonstrated that, in some cases, changes in blood sugar levels are accompanied by refractive change. We note, however, that in such patients the blood sugar changes may be much larger than in our work and that the refractive changes may occur over much longer timescales than a single day. It may also be that fluctuations in vision in at least some diabetic patients are caused by factors other than refractive error fluctuations.
